# Supplementary figures and images for: Integrative genomic characterization of five Pediococcus acidilactici strains reveals differing probiotic safety profiles
Source: PLoS One. 2025 Sep 30;20(9):e0332506. doi: 10.1371/journal.pone.0332506 (PMC12483243; doi:10.1371/journal.pone.0332506)

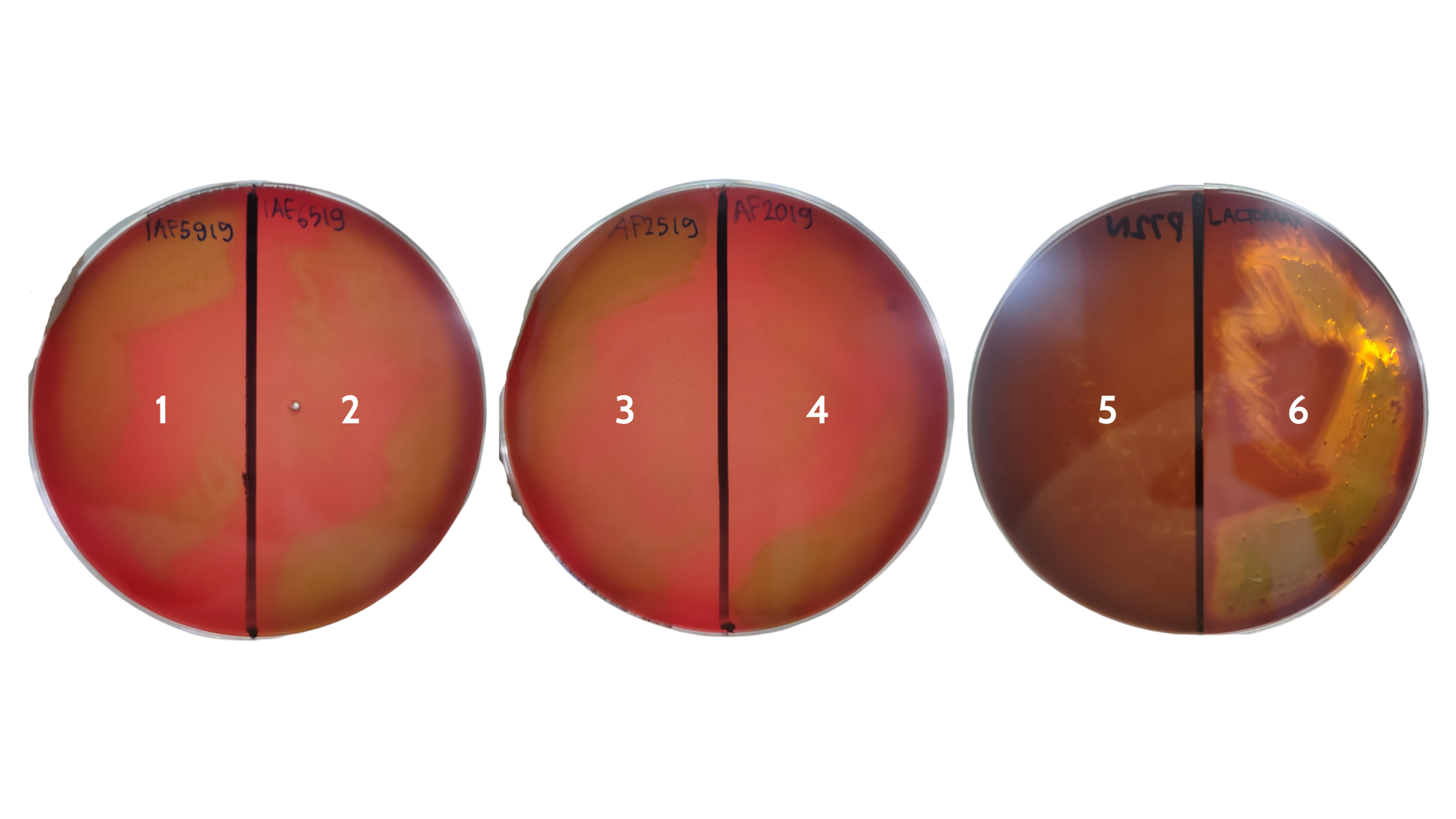

Supplement: S1 Fig — 1: IAF5919; 2: IAF6519; 3: AF2519; 4: AF2019; 5: P72N; 6: Bacillus sp. (TIF) [file pone.0332506.s003.tif]

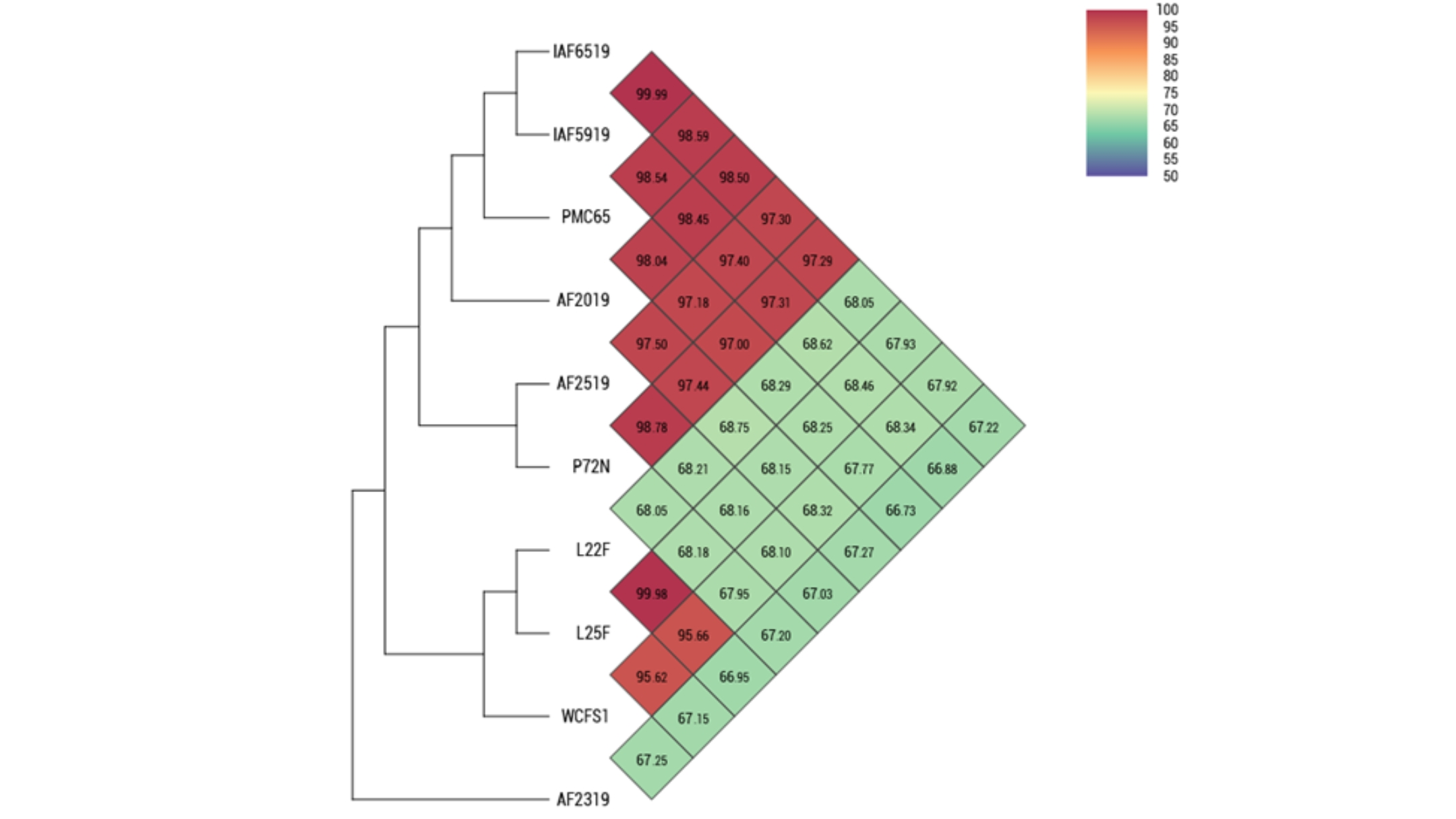

Supplement: S2 Fig — All five Thai strains shared >97% ANI with the reference P. acidilactici strain PMC65, confirming species identity. In contrast, ANI values between these and strains such as WCF51, L25F, and AF2319 were substantially lower (<70%), supporting their taxonomic distinction and reaffirming the correct species-level classification. (TIF) [file pone.0332506.s006.tif]

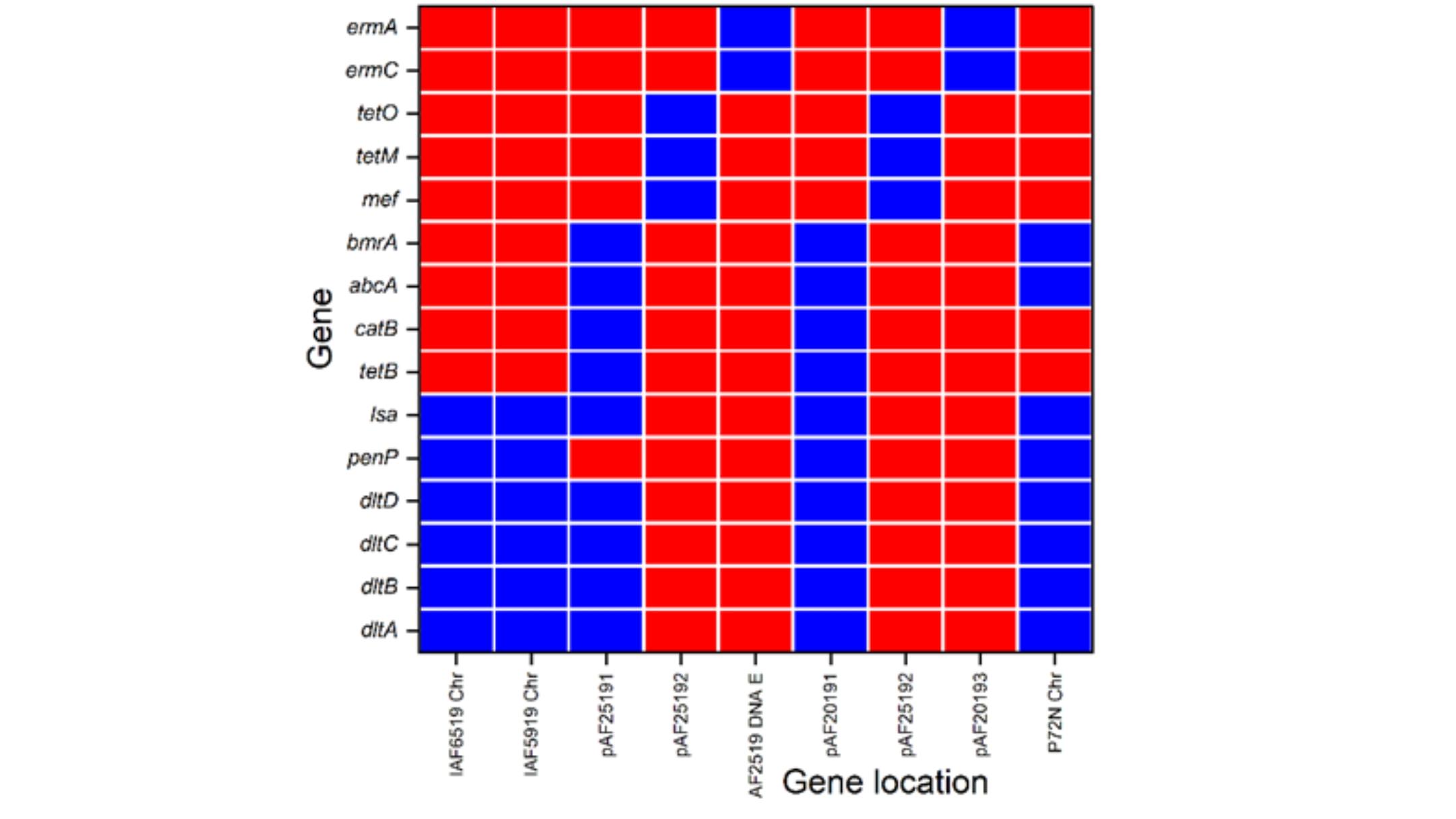

Supplement: S3 Fig — Blue indicates the presence of the virulence factor genes, while red indicates their absence. (TIF) [file pone.0332506.s007.tif]
