# Supplementary material for: Integrative genomic characterization of five Pediococcus acidilactici strains reveals differing probiotic safety profiles
Source: PLoS One. 2025 Sep 30;20(9):e0332506. doi: 10.1371/journal.pone.0332506 (PMC12483243; doi:10.1371/journal.pone.0332506)
Supplement: Attachment — Submitted filename: Response to Reviewers.docx [file pone.0332506.s018.docx]

Rebuttal Notes

Dear Editor

We thank the reviewer for the positive assessment of our manuscript and the constructive comments that have helped us to improve the clarity and contextualization of our findings. We have addressed each point as detailed below, with changes highlighted in the “Revised Manuscript with Track Changes” file.

Comment 1: Abstract: "novel composite alignment" of tet(M); this terminology could be explained more precisely to aid the general readership

Response: In the revised abstract, we have replaced the term *“novel composite alignment”* with a more precise and descriptive phrase: *“novel composite genetic arrangement flanked by mobile elements, suggesting historical recombination and altered mobility potential.”* This change clarifies the genetic context of *tet(M)*, indicating its integration within a composite structure bounded by mobile genetic elements and providing insight into its potential evolutionary origin and mobility. This revision aims to improve clarity for the general readership and to better convey the biological significance of our finding.

Change location: Abstract, lines 37–38.

Comment 2: Discussion: The observed susceptibility to tetracycline in the presence of tet(M) and resistance to clindamycin in the absence of comparable ARGs warrants further mechanistic discussion. Could gene silence, promoter inactivity, or undiscovered resistance factors be involved? Please discuss more about it.

In the revised *Discussion* (page XX, paragraph XX), we have expanded the explanation to include possible mechanisms for tetracycline susceptibility despite *tet(M)*, such as promoter inactivity, structural mutations, and inducible expression. For clindamycin resistance without *erm* or *lnu* genes, we now discuss potential roles of 23S rRNA or ribosomal protein mutations, overexpression of MFS efflux pumps, and uncharacterized determinants such as *cfr*. These additions provide a clearer rationale for the observed genotype–phenotype discrepancies.

Change location: Discussion, lines 412–431.

Comment 3: Discussion: The discovery of katA and cfa, which are rarely seen in P. acidilactici, is interesting. These findings indicate potential innovative adaptations. Please find literature or brief contextualization to discuss.

Response: we now describe *katA* as a rare heme-dependent catalase in LAB, potentially acquired from catalase-positive gut or feed bacteria, enhancing oxidative stress tolerance and survival in aerobic and gastrointestinal conditions. *Cfa* encodes cyclopropane fatty acid synthase, which increases membrane rigidity and hydrophobicity, improving acid and bile tolerance, stability during feed processing, and intestinal persistence. Together, these genes may represent an adaptive signature that enhances resilience for livestock probiotic applications.

Change location: Discussion, lines 457–470.

S1 Fig. Hemolytic Activity Test of five Thai P. acidilactici strains: If you have a clearer image, please replace it for plate marked no. 5 and 6

Response: We have replaced the original S1 Fig. with a high-resolution TIFF image showing plates 5 and 6 more clearly. The updated image improves visibility of hemolysis zones, enabling clearer distinction of alpha-hemolysis. The figure legend has been retained but the image quality now meets PLOS ONE resolution standards (≥300 dpi).

Change location: Supplementary Figure S1.

Editorial Requirement 1: Data Availability Statement

We have revised the Data Availability Statement as requested. The accession numbers for all genome assemblies have been included, along with the repository name (NCBI GenBank) and the link to the database. Additional annotations are provided in the Supplementary File.

Change location: Data Availability Statement, lines 536-542

Editorial Requirement 2: Figure Captions

All figure captions are now included separately in the manuscript following the PLOS ONE format, placed immediately after the first mention of the figure in the text.

Editorial Requirement 3: References

The reference list has been checked and updated to ensure accuracy. No retracted papers are included.

Response to Editorial Requests

PLOS ONE formatting: Manuscript reformatted per PLOS templates.

Data Availability Statement: Revised to include repository names and GenBank accession numbers in the correct format.

Figure captions: Added standalone captions for all figures.

Reference list: Checked for completeness, accuracy, and currency. No retracted articles are cited. New references added for katA, cfa, and resistance mechanism discussion.

We believe these revisions have improved the clarity, completeness, and impact of our manuscript, and we look forward to your further consideration.

On behalf of all authors,

Nuvee Prapasarakul

Corresponding author
